# Downregulation of circ-Foxo3 in breast cancer stem-like cells

**DOI:** 10.1186/s13104-023-06405-z

**Published:** 2023-07-03

**Authors:** Mohammad Kamalabadi-Farahani, Amir Atashi, Mohammad Masoud Eslami

**Affiliations:** 1 Department of Tissue Engineering, School of Medicine, Shahroud University of Medical Sciences, Shahroud, Iran; 2 Department of Medical Laboratory Sciences, School of Allied Medical Sciences, Shahroud University of Medical Sciences, Shahroud, Iran; 3 Department of Hematology, School of Medicine, Tarbiat Modares University (TMU), Tehran, Iran

**Keywords:** Breast Cancer, Circular RNA, Cancer Stem cells, Spheroids, Circ-Foxo3

## Abstract

**Objective:**

Cancer cells having stem cell characteristics are linked to metastasis and relapse in breast cancer. Circ-Foxo3, as a circular RNA, has been linked to the breast cancer lethal traits. This study’s objective was to assess circ-Foxo3 expression in breast cancer stem-like cells. After isolation from tumor mass, breast cancer cells were subjected to the reliable in vitro assay of spheroid formation to determine the presence cancer stem cells (CSCs). We used a quantitative real-time polymerase chain reaction to examine circ-Foxo3 expression in spheroids.

**Results:**

Circ-Foxo3 expression was significantly downregulated in spheroid-forming tumor cells, according to our data. This study demonstrated that breast CSCs have downregulated circ-Foxo3 expression, which may allow these cells to evade apoptosis. A precise analysis of this circRNA’s role could be exploited to develop focused therapeutic approaches to fight breast CSCs.

## Introduction

Worldwide, breast cancer is the most prevalent malignancy in women [1]. Recurrence and metastasis are likely lethal events that happen in breast cancer patients [2]. The existence of a rare subset of stem cells among heterogeneous populations of cancer cells, particularly breast cancer, has been made clear in recent decades. These unique cells, sometimes referred to as cancer stem cells or cancer-initiating cells, are characterized by the presence of surface biomarkers, multi-drug resistance pumps, and aberrant self-renewal pathways. They play a crucial part in promoting the growth of tumors and the progressive spread of cancer cells [3].

When cultivated in specific culture conditions, CSCs have been shown to have the capacity to form spheres in vitro [4]. The best method for identifying cancer stem cells is to use these sphere-forming cells, which are used as a standard experimental test for assessing the possibility of stemness in cancer cells [5]. Tumor-derived multicellular spheroids are purposed for the enrichment of cancer stem cells (CSCs) or cells with stem cell-related characteristics. These spheroids are grown as floating spheres and have been used as surrogate systems to evaluate the CSC-related characteristics of solid tumors in vitro [6].

Circular RNAs (circRNAs), a novel family of non-coding RNAs (ncRNAs), control the translation of proteins or peptides, downstream gene expression, and linear RNA transcription, all of which are essential for many biological activities. Recent research in the field of tumor biology has indicated that circRNAs have the ability to be either oncogenic or anti-oncogenic and play crucial regulatory functions in the development and spread of tumors [7].

One of the circRNAs that has been extensively researched in relation to cancer is circular RNA Forkhead box O3 (CircFoxo3). It comes from the FOXO3 gene and controls cell cycle, cell apoptosis, and stress resistance. A tumor suppressor gene, Foxo3 protein is downregulated in various tumors [8]. CircFoxo3, depending on the environment in which cancer is being evaluated, has shown both tumor suppressor and oncogenic potential. By raising the level of the Foxo3 protein and suppressing the expression of p53 in breast cancer, circFoxo3 functions as a tumor suppressor. As a result, overexpression of the Foxo3 downstream targets causes cell apoptosis [9].

Circ-Foxo3 has been linked to the growth and carcinogenesis of a number of malignancies, including breast cancer, according to earlier research. In this regard, the goal of the current work was to examine the expression of this circular RNA in mouse breast cancer cells and the relationship between these cells and the cancer stem-like cells found in multicellular breast cancer spheroids.

## Materials and methods

### Cell culture

4T1 cell line was obtained from the cell bank of Pasteur Institute of Iran (C604). The cells were cultured in high glucose Dulbecco’s Modified Eagle’s Medium (DMEM) containing 10% FBS (fetal bovine serum) and 2% Penicillin-Streptomycin (all from Gibco, USA) in humidified atmosphere of 5% CO2 at 37 °C.

### Breast tumor induction and isolation of heterogeneous population of cancer cells

Mammary tumors induction and isolation of a heterogeneous population of tumor cells were performed as described in our previous study [10]. Briefly, female BALB/c mice weighing 20–25 g were obtained from the Royan Institute (Iran). The animals were kept in cages at 12 h photoperiod while they had free access to food and water. The ethics committee of Shahroud University of Medical Sciences approved this study for ethics in animal research (registration number: IR.SHMU.REC.1400.265). Subcutaneous injection of 4T1 cells (1 × 10^5^) was performed into the mice’s flank (or the right hind limb). For isolation of a heterogeneous population of tumor cells, tumors were excised from mice after 35 days of tumor induction. Mincing and enzymatical digestion of tumor tissue were performed in aseptic conditions. The digested tumor was filtered through 70 μm cell strainers and cultured in a DMEM with 10% FBS and 2% Penicillin–Streptomycin (all from Gibco, USA). The cells were ultimately incubated at 37 C in 5%CO2 and passaged twice.

### 2D monolayer and 3D multicellular spheroids generation

The following procedures were followed to produce and culture a diverse population of cancer cells that had been isolated in earlier steps as a 2D monolayer and 3D multicellular suspension:

1- For the 2D monolayer, cells were plated in 96-well culture plates at a density of 1 × 10^4^ cells per well and grown in high glucose DMEM with 10% FBS and 2% Penicillin-Streptomycin (both from Gibco, USA) at 37 °C in a humidified environment of 5% CO2.

2-CSCs are a small subset of the cancer cells among a heterogeneous population of cancer cells. Accordingly, it is necessary to isolate CSCs from the rest of the cancer cells. The spheroids are purposed for the enrichment of cancer stem cells (CSCs) or cells with stem cell-related characteristics. To create 3D spheroids, cells were plated in a 96-well Ultra-Low-Attachment Microplate at a density of 1 × 10^4^ cells per well, cultured in spheroid-forming media made of high glucose DMEM containing 0.5% FBS and 2% Penicillin-Streptomycin (all from Gibco, USA), and incubated for 6 days at 37 °C in a humidified atmosphere of 5% CO2.

### Quantification of circ-Foxo3 by RT-qPCR

After 48 h Total RNA was extracted from both 2D monolayer and 3D spheroids using QIAzol Lysis Reagent (QIAGEN). The quality, yield, and size of extracted RNA were analyzed using spectrophotometry (NanoDrop-ThermoFisher) and electrophoresis. The first strand cDNA synthesis was performed using reverse transcription system (Easy cDNA Synthesis Kit for RNA or mRNA to cDNA - pars tous). Real-time PCR procedure was executed based on the 1 ul cDNA in all samples. Quantization of all gene transcripts was done by SYBR Green Real time PCR Master Mix (Amplicon A/S, Denmark) using StepOnePlus™ Real-Time PCR System, according to the manufacturer’s instruction. The amplification procedure was as follows: 1 cycle of 95 °C for 15 min, 40 cycles of 95 °C for 30 s, 60 °C for 30 s, and 72 °C for 30 s. The exact mRNA expression was normalized to the expression level of GAPDH. Relative changes of gene expression were calculated by the following formula, and the data was represented as fold up-regulation/down-regulation.

Fold change = 2^−ΔΔCt^, where ΔΔCt = [Ct of circ-Foxo3 (in 3D spheroids) - Ct of GAPDH (in 3D spheroids)] - [Ct of circ-Foxo3 (in 2D monolayer) - Ct of GAPDH (in 2D monolayer)].

Primers were designed using AlleleID version 6 software (Premier Biosoft Inc.).

The used primers are as follows:

For circ-Foxo3.

forward 5′-AAATGGGCAAAGCAGAACTC-3′, reverse 5′- TCGGCTCTTGGTGTACTTG − 3′.

For GADPH.

forward 5′-CCTGGAGAAACCTGCCAAGTA-3′, reverse 5′-GGCATCGAAGGTGGAAGAGT − 3′.

### Statistical analysis

Results are expressed as the mean ± standard deviation. Data were analyzed with GraphPad Prism statistical software 6.0 (GraphPad Software, La Jolla, CA, USA) using Paired Samples t Test. P < 0.05 was considered statistically significant.

## Results

### Isolation of heterogenous population of tumor cells

**Fig. 1:**
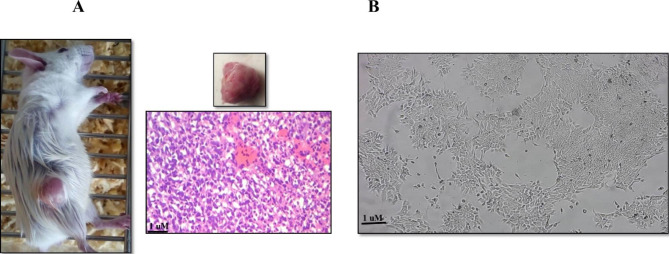
Primary Tumor Cells Isolation. **(A)** Metastatic animal model of triple negative breast cancer was generated after 20 days of tumor induction in Balb/c mice. **(B)** Primary tumor isolation, H&E staining and primary tumor cell extraction was performed on primary tumor tissues. Magnification 40x. Scale bar 1 μm

Due to multiple passages and manipulations, most breast cancer cell lines have changed their function and genome [11]. So, we decided to use the heterogeneous population of primary tumor cells to create spheroid. For this, animal model of triple negative breast cancer was generated (Fig. 1A). When tumor mass is palpable (20 days following tumor induction in Balb/c mice) tumor mass was removed aseptically (Fig. 1B). H and E staining and pathological confirmation were performed on tumor tissues (Fig. 1B). Heterogenous population of tumor cells was isolated from tumor mass with enzymatical and mechanical digestion (Fig. 1B).

### Multicellular breast cancer spheroids formation

**Fig. 2:**
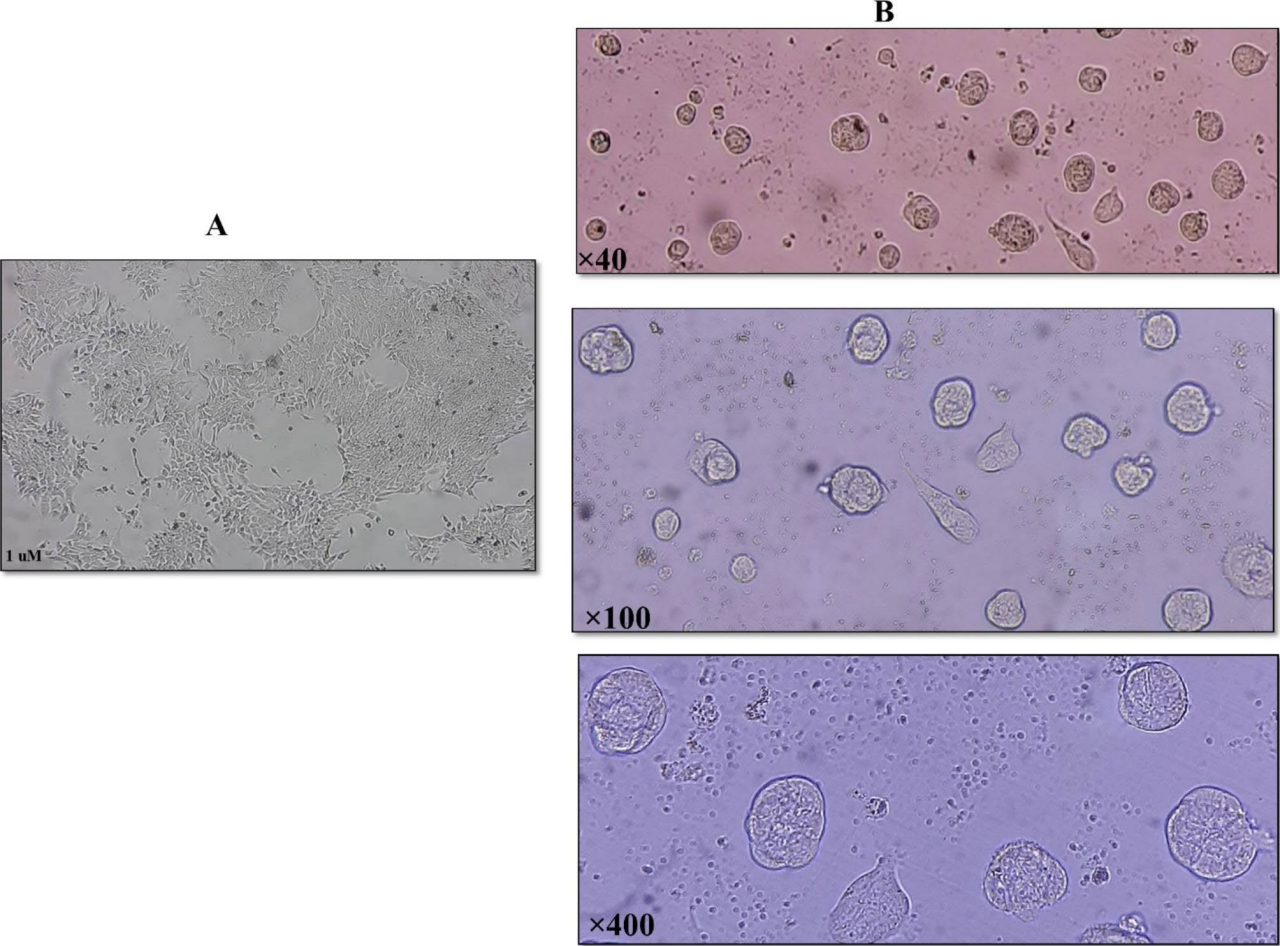
Spheroid Conduction in Heterogenous Population of Tumor Cells. **(A)** 2D cell culture of heterogenous population of tumor cells isolated from primary tumor mass. **(B)** Multicellular breast cancer spheroids generated from heterogenous population of tumor cells in special culture condition after 6 days. Magnification 40x, 100x and 400x. Scale bar 1 μm

For spheroid formation among heterogenous population of tumor cells, we used non-adherent 96 well plates. As shown in the Fig. 2, after 6 days, the spheroids formed in the well. At this stage, the spheroids were ready for treatment with the extract.

### Significant down-regulation of circ-Foxo3 in spheroids forming tumor cells

**Fig. 3:**
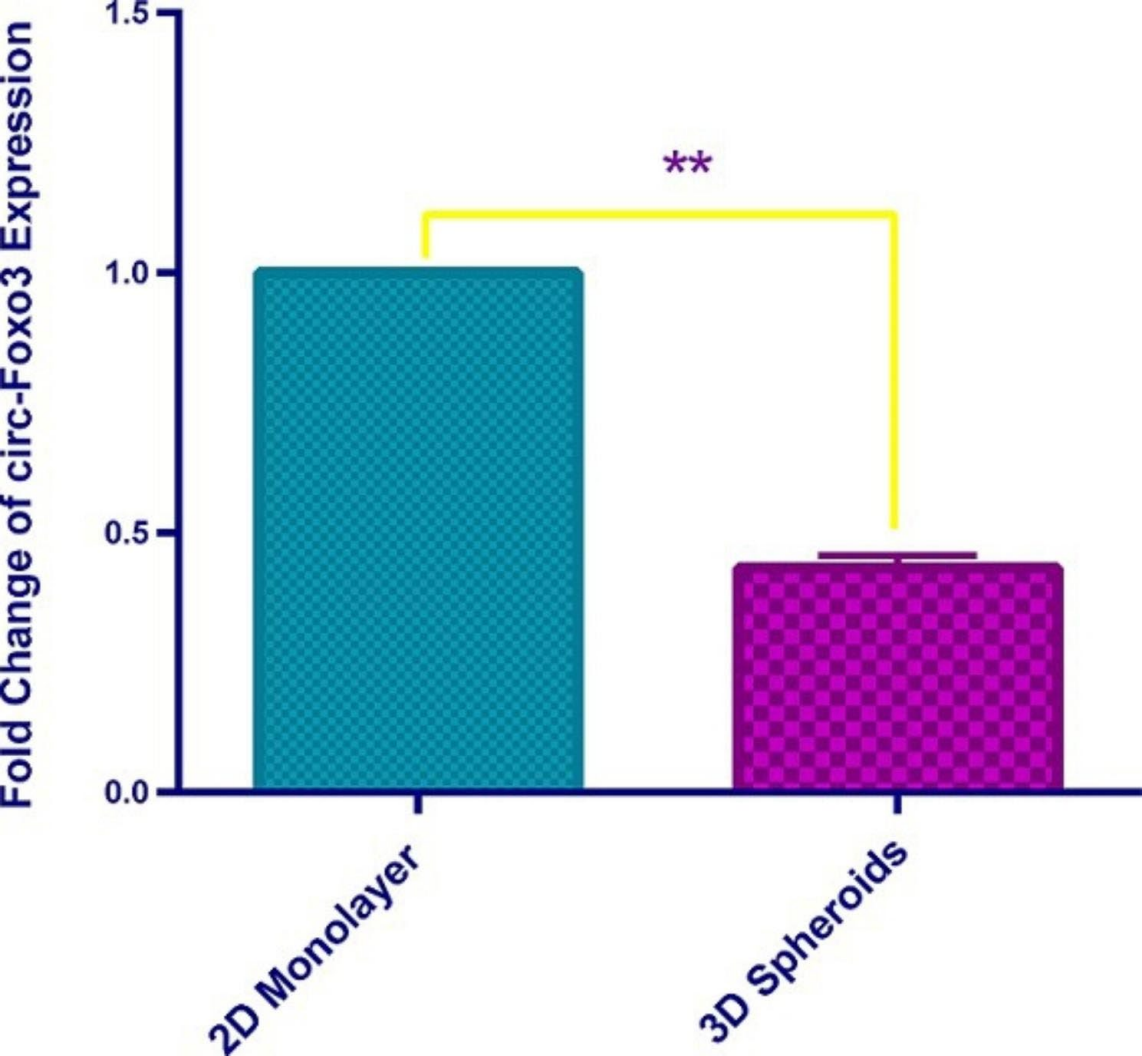
Expression of Circ-Foxo3 in Multicellular Breast Cancer Spheroid Using Real-Time PCR. Circ-Foxo3 was significantly downregulated in multicellular breast cancer spheroids. All results are expressed as mean ± SD from at least three independent experiments on 3 samples, analyzed by Two-tailed T test. **P < 0.001

The expression of circ-Foxo3 was analyzed in tumor cells both in 2D monolayer and 3D multilayer. The quality, yield, and size of extracted RNA, synthesized cDNA, and PCR products were confirmed using NanoDrop and gel electrophoresis. As shown in Fig. 3 expression of circ-Foxo3 was significantly down regulated in spheroid-forming tumor cells. Compared with breast tumor cells in 2D monolayer, circ-Foxo3 was downregulated 2.3 time in multicellular breast cancer spheroids.

## Discussion

In the current work, we found that multicellular breast cancer spheroids have significantly less circ-Foxo3 expression. Our results indicate that Circ-Foxo3 expression has been grossly altered in these multicellular spheroids that contain breast cancer stem-like cells. This is the first account we are aware of the characterization of these molecules in breast cancer stem cells.

CircRNAs are unregulated in various types of human malignancies, including breast tumors, according to an increasing number of research published in recent years [12]. In 2016, Yang et al. demonstrated that ectopic expression of the circ-Foxo3 may inhibit the growth of tumors and the proliferation of cancer cells. They demonstrated that ectopic expression of circ-Foxo3 drastically reduced cell proliferation and survival of the MDA-MB-231 breast cancer cell line. In a mouse model, it was demonstrated that circ-Foxo3 transfected MDA-MB-231 tumor growth considerably decreased as compared to the control [13]. Unregulated expression of Circ-Foxo3 in breast CSCs is the main finding of our work.

Circ-Foxo3 is essential for the apoptosis of breast cancer cells, according to Du et al. works. They demonstrated that circ-Foxo3 was weakly expressed in both patient tumor samples (relative to benign tissue) and a panel of breast cancer cell lines (compared to normal cell lines). It’s interesting to note that circ-Foxo3 expression was discovered to be considerably higher during cancer cell death [9]. In our previous works apoptotic resistance of breast CSCs have been confirmed [14]. Accordingly down regulation of Circ-Foxo3 in breast CSCs may be a reason for apoptotic resistance of these cells. We argued that by down regulating circ-Foxo3, breast CSCs can evade apoptosis because of the crucial role that circ-Foxo3 plays in the apoptosis of breast cancer cells.

Studies on the functions of circular RNAs in CSCs are scarce. In laryngeal squamous cell carcinoma [15], hepatocellular carcinoma [16], bladder cancer [17], and breast cancer, the roles of Circ0005033, CircumMALAT1, CircRNAs 103,809 and CircSKA3 [18] in cancer stem cell have been detected respectively. For first time, the role of circ-FOXO3 in breast CSCs are discussed and emphasized in our work.

In addition to providing potential diagnostics and targets for cancer patients, the study of circRNAs in the regulation of CSCs would bring new insights into the molecular mechanisms of CSCs. To the best of our knowledge, no research has been done on the function of circ-Foxo3 in CSCs. This is the first account of these compounds’ characterization in breast cancer stem cells. This work demonstrated that breast CSCs have downregulated circ-Foxo3 expression, which suggests that these cells may be able to avoid apoptosis in this fashion. A precise analysis of this circRNA’s role could be exploited to develop focused therapeutic approaches to fight breast CSCs. We still need to learn more about the expression and significance of circ-Foxo3 and other endogenous circular RNAs, both in vitro and in vivo.

### Limitation

Designing a specific primer set for circ-FOXO3 is the major limitation of this project because this RNA has a very complicated structure.

## Data Availability

The datasets used and/or analyzed during the current study available from the corresponding author on reasonable request.
